# Information Literacy Assessment with a Modified Hybrid Differential Evolution with Model-Based Reinitialization

**DOI:** 10.1155/2018/9745639

**Published:** 2018-10-22

**Authors:** Yuan Wang, Hui Li, Zhenguo Ding

**Affiliations:** ^1^Faculty of Information Engineering, China University of Geosciences (Wuhan), No. 388 LuMo Road, Hongshan District, Wuhan, China; ^2^School of Computer, China University of Geosciences (Wuhan), No. 388 LuMo Road, Hongshan District, Wuhan, China; ^3^Institute of Higher Education, China University of Geosciences (Wuhan), No. 388 LuMo Road, Hongshan District, Wuhan, China

## Abstract

Information literacy assessment is extremely important for the evaluation of the information literacy skills of college students. Intelligent optimization technique is an effective strategy to optimize the weight parameters of the information literacy assessment index system (ILAIS). In this paper, a new version of differential evolution algorithm (DE), named hybrid differential evolution with model-based reinitialization (HDEMR), is proposed to accurately fit the weight parameters of ILAIS. The main contributions of this paper are as follows: firstly, an improved contraction criterion which is based on the population entropy in objective space and the maximum distance in decision space is employed to decide when the local search starts. Secondly, a modified model-based population reinitialization strategy is designed to enhance the global search ability of HDEMR to handle complex problems. Two types of experiments are designed to assess the performance of HDEMR. In the first type of experiments, HDEMR is tested and compared with seven well-known DE variants on CEC2005 and CEC2014 benchmark functions. In the second type of experiments, HDEMR is compared with the well-known and widely used deterministic algorithm DIRECT on GKLS test classes. The experimental results demonstrate the effectiveness of HDEMR for global numerical optimization and show better performance. Furthermore, HDEMR is applied to optimize the weight parameters of ILAIS at China University of Geosciences (CUG), and satisfactory results are obtained.

## 1. Introduction

With the arrival of the new economic era based on information and knowledge, information has become an important factor in all fields of society. Information literacy (IL) skills which are the ability to locate, evaluate, and effectively use the needed information have become more important, especially to college students. At present, there were some college education information literacy standards such as the American Association of College and Research Libraries (ACRL) standard, including 5 first-level indexes, 22 second-level indexes, and 86 third-level indexes, the Australian and New Zealand Institute of Information Literacy (ANZIIL) standard consisting of 6 first-level indexes, 19 second-level indexes, and 67 third-level indexes, and the Society of College, National and University Libraries (SCONUL) standard in the United Kingdom which was composed of 7 first-level indexes and 17 second-level indexes. Nevertheless, in the face of the explosion of such digital information, it is difficult for college students to obtain the required information accurately.

During the last few decades, information literacy has attracted much attention by the researchers. Webber and Johnston [1] identified some key definitions of information and related the student response to two models of information literacy. Cooney and Hiris [2] described the use of a collaborative framework for integrating information literacy into a graduate finance course and used a checklist for assessing the results. Valerie et al. [3] proposed the revised assessment method that took the form of a portfolio and reported the results of a case study evaluating the revision of the assessment methods of an information literacy module. The results showed that it was also economical and efficient. Pinto [4] designed the IL-HUMASS survey on information literacy, and it contained 26 items grouped into four categories and three self-reporting dimensions. Naimpally et al. [5] introduced a broad overview of the different assessment tools in information literacy assessment and highlighted four assessment tools for assessment of IL in engineering. Kousar and Mahmood [6] assessed IL skills of first year undergraduate engineering students at a Pakistani university in order to plan instruction. They used independent samples *t*-test and ANOVA to analyze the reliable data for integration of instruction in the university curricula. Krakowska [7] gathered the data from the questionnaire and analyzed it briefly in a quantitative and qualitative manner. The evaluation of the results helped to understand the students' information behavior, increased awareness of information literacy implementation, and development and status within the academic information environment. Pavlovski and Dunđer [8] presented results of a survey regarding information literacy which was carried out on undergraduate students of University of Zagreb and gained an insight into the students' information retrieval accuracy in course-related research. Kultawanich et al. [9] reported on the development and validation of the information literacy assessment tools for undergraduate students. These assessment tools consisted of three instruments: (1) IL Test, (2) IL Rubric, and (3) Information Literacy Self-Efficacy (ILSE) Scale. Douglas et al. [10] presented the development and initial validation study of a self-directed information literacy assessment for engineering and technology students. The exploratory factor analysis results provided evidence of structural aspects of validity and support for scoring structure. Kavšek et al [11] used two groups of first year psychology students to evaluate the effect of information literacy (IL) training in the first year psychology students and to follow changes in acquired IL in time. The results had shown an important role of IL training in students' IL development over time. Lanning and Mallek [12] analyzed multiple factors from current university students' high school experiences to evaluate the students' information literacy skills. The results of regression analyses demonstrated that only current university GPA and standardized test scores had any influence on information literacy test scores.

An optimization problem is the problem of finding the best solution from all feasible solutions. Optimization problems can be divided into two categories depending on whether the variables are continuous or discrete. The standard form of an optimization problem is formulated as

(1) $$\begin{array}{c} \underset{x\in D_{0}}{\min}\;f\left(x\right),\\ D=\left\{x\in D_{0}\left|f_{j}\right.\left(x\right)\leq 0,\;j=1,\ldots ,J\right\}, \end{array}$$

where *D*_0_={*x* ∈ *E*^*n*^|*ℓ* ≤ *x* ≤ *u*} is a simple box constraint set. Problem category included discrete domain and continuous domain. Optimization approaches included deterministic search methods (such as direct search methods, simplex-based search, and branch and bound search methods) and nondeterministic (heuristic) search methods (such as simulated annealing, genetic algorithms, differential evolution, and particle swarm optimization).

In the deterministic search methods, the researchers proposed the global optimization methods that can tackle multimodal optimization problems satisfying the Lipschitz condition [13]. Sergeyev and Kvasov [14] proposed a new efficient algorithm for solving the multidimensional “black-box” functions satisfying the Lipschitz condition. In this algorithm, a novel technique balancing usage of local and global information during partitioning, and a new procedure for finding lower bounds of the objective function over hyperintervals were considered. Jones et al. [15, 16] proposed the well-known and widely used algorithm DIRECT (DIviding RECTangles) which objective function was evaluated at several sample points by using all possible weights on local versus global search expressed by the Lipschitz constant during each iteration. Liuzzi et al. [17] focused on the selection strategy and proposed two strategies to exploit the information on the objective function. The first one was based on the knowledge of the global optimum value of the objective function. The second one did not require any a priori knowledge on the objective function and tried to exploit information on the objective function gathered during progress of the algorithm. Paulavičius et al. [18] proposed a globally biased simplicial partition DISIMPL algorithm for global optimization of expensive Lipschitz continuous functions to improve the search efficiency of the algorithm. Gimbutas and Žilinskas [19] introduced a bi-criteria choice of simplices for the subdivision to enhance the efficiency of the simplicial partition-based minimization of black box objective functions. The first criterion was minimum of an estimated Lipschitz bound, and the second was the size of the candidate simplex. Sergeyev et al. [13] proposed a new efficient visual technique for a systematic comparison of global optimization algorithms and tried to bridge the gap between the deterministic methods and heuristic methods.

In the heuristic search methods, DE and its variants have emerged as one of the most competitive and versatile families of evolutionary algorithms which belong to computational intelligence. It was first proposed by Storn and Price [20] to solve global numerical optimization problems over continuous search spaces. It is a simple yet powerful evolutionary algorithm and exhibits excellent capability in solving a variety of numerical and real-world optimization problems, such as space trajectory design [21–25], hydrothermal optimization [26], underwater glider path planning [27], vehicle routing problem [28, 29], short-term optimal hydrothermal scheduling [30], satellite scheduling [31, 32], and satellite image enhancement [33].

During the last decade, there were many researchers working on the improvement of DE and significant progress in the developments of DE. In 2011 and 2016, Das et al. [34, 35] presented a comprehensive survey on DE, including the basic concepts and major variants of DE, as well as the applications and theoretical studies of DE. Next, we will briefly introduce the recent developments of hybrid DE in the last two years.

Hybrid DE was tried to combine DE with other global or local search techniques. Awad et al. [36] introduced a novel hybridization between differential evolution and update processes of the stochastic fractal search algorithm. Ali et al. [37] and Awad et al. [38], respectively, focused on the development of hybridizing cultural algorithm (CA) with DE. The purpose was to combine the explorative and exploitative capabilities of two evolutionary algorithms. Cai et al. [39] proposed an adaptive social learning (ASL) strategy for DE, named SL-DE, to extract the neighborhood relationship information of individuals in the current population. Jadon et al. [40] proposed a hybridization of artificial bee colony (ABC) and DE algorithms, called HABCDE, to develop a more efficient meta-heuristic algorithm than ABC and DE. Peng et al. [41] hybridized DE with commensal learning and uniform local search (CUDE). In CUDE, commensal learning was proposed to adaptively select optimal mutation strategy and parameter setting simultaneously under the same criteria. Moreover, uniform local search enhanced exploitation ability. Zhao et al. [42] proposed a hybrid optimization algorithm based on chaotic differential evolution and estimation of distribution (cDE/EDA). The proposed algorithm could discover the optimal solution in a fast and reliable manner. Chaotic policy was used to strengthen the search ability of DE. Peng et al. [43] proposed an improved memetic differential evolution algorithm, called MDE, which hybridized differential evolution with a local search (LS) operator and periodic reinitialization to balance exploration and exploitation for solving global optimization problems. Under the framework of LSHADE in [44], Mohamed et al. [45] further proposed a new semi-parameter adaptation approach to effectively adapt the values of the scaling factor, named LSHADE-SPA. And then, a hybridization framework named LSHADE-SPACMA, a combination of LSHADE-SPA and a modified version of CMA-ES, were introduced. Sabar et al. [46] proposed a heterogeneous framework that integrated a cooperative coevolution method with various types of memetic algorithms for solving big data optimization problems. In this heterogeneous framework, DE was utilized to optimize the subproblems generated by the cooperative coevolution method.

This work is motivated by the following observations:

1. The main difficulties in college students' information literacy assessment index system are the logical structure of the index system and how to assign weight parameters to the index system. There are mainly two ways to determine weights: one is subjective assignment, and the other is objective assignment. Subjective assignment mainly refers to determining weights according to the human experience and knowledge. Objective assignment is based on the data of questionnaire. Hence, it is very necessary for ILAIS to use advanced optimization techniques to fit weight parameters.
2. The investigations in [21–33] indicate that DE has been successfully used in a variety of domains. However, the use of DE for the weight parameters determination of ILAIS has not been reported.
3. As a kind of intelligent optimization techniques, DE produces relatively good results in different fields. However, DE has been demonstrated to converge to a fixed point, a level set [23] or a hyperplane not containing the global optimum [47]. Furthermore, in some cases it is shown to have slow local convergence. Thus, integrating additional strategies to improve DE optimization performance is worth investigating.

Based on the above considerations, in this paper, we propose a modified hybrid DE, called HDEMR, with the local search algorithm Broyden–Fletcher–Goldfarb–Shanno (BFGS) to improve local convergence. A modified contraction criterion based on the entropy of population in objective space and the maximum distance in decision space is employed to trigger local search. In addition, we define an archive to save the best individual in the population after the local search. And then, the population is reinitialized based on the probability model of the archive.

The paper is organized as follows: in Section 2, the college students' information literacy assessment index system and the weight parameter optimization model of ILAIS are introduced. DE is briefly introduced in Section 3. The proposed algorithm is described in details in Section 4. The experimental study and results analysis are presented in Section 5. HDEMR for weight parameter optimization of ILAIS is derived in Section 6. Finally, in Section 7, we conclude this paper and suggest ideas for future work.

## 2. The Weight Parameter Optimization Model of ILAIS

For the sake of completeness, in this section, the college students' ILAIS at China University of Geosciences (CUG) is briefly described first. Then, the weight parameter optimization model of ILAIS based on an objective function is introduced.

### 2.1. The Establishment of ILAIS at CUG

Based on the new requirements of education and college students' feature, ILAIS at CUG is established into four categories: Information Consciousness, Information Knowledge, Information Ability, and Information Morality. We treat these four categories as first-level indexes. Then, for reference-related standards such as ACRL, we gradually decompose first-level indexes and initially build thirteen second-level indexes of ILAIS at CUG. Finally, the framework of ILAIS at CUG includes four first-level indexes and thirteen second-level indexes, which is shown in Table 1.

### 2.2. The Weight Parameter Optimization Model

Before using optimization techniques to identify the weight parameters of the ILAIS at CUG, there are two issues that must be addressed. First, the weight parameters that will be optimized must be chosen. In the above ILAIS at CUG established in Table 1, the number of weight parameters of ILAIS at CUG is related to the level. To first-level index, there are four weight parameters, and to second-level indexes, there are thirteen weight parameters.

In this work, all weight parameters are treated as unknown parameters to be identified by the optimization algorithm. Thus, the decision vector *x* is formulated as

(2) $$\begin{array}{c} x=\left\{x_{1},x_{2},\ldots ,x_{D}\right\}, \end{array}$$

where *D* is the number of decision variables. An example for the determination of the first-level index weight parameters is given here. Let the weight of Information Consciousness be *x*_1_, the weight of Information Knowledge be *x*_2_, the weight of Information Ability be *x*_3_, and the weight of Information Morality be *x*_4_.

The other important issue in evolutionary algorithms is to determine an objective function. When optimization techniques are used in parameter identification problems of ILAIS, the objective function should be defined first. In this work, the weight deviation sum is used as the objective function in the following equation:

(3) $$\begin{array}{c} \min f\left(x\right)=t\;*\;\overset{N_{p}}{\underset{i=1}{\sum }}\overset{P}{\underset{p=1}{\sum }}\left(\left|x_{p,j}-x_{i,j}\right|\right)+s\;*\;\overset{N_{p}}{\underset{i=1}{\sum }}\overset{Q}{\underset{q=1}{\sum }}\left(\left|x_{q,j}-x_{i,j}\right|\right), \end{array}$$

where *P* and *Q* are, respectively, the number of teachers and students participating in the questionnaire. *t* and s are the authority coefficient of teachers and students. *x*_*p*,*j*_ is the weight vector given by the *p*-th teacher and *x*_*q*,*j*_ is the weight vector given by the *q*th student. *x*_*i*,*j*_ is the *i*-th weight individual, *N*_*p*_ is the population size, and *j*=1,2,…, *D* in HDEMR.

## 3. A Short Introduction to Differential Evolution

DE is a population-based stochastic parallel direct search optimization method. It begins with a randomly generated population in decision space. Then, DE iteratively uses a trial vector generation strategy (i.e., mutation and crossover operators) and selection operator to evolve the population until a stopping criterion is met.

For a mutation operator, there are five frequently used mutation schemes for generating a mutant vector. It can be denoted as DE/*a*/*b*, where *a* stands for the mutation strategy and *b* specifies the number of difference vectors. The following are the five most frequently used mutation strategies:

(4) $$\begin{array}{c} \text{“DE}/\mathrm{rand}/1\text{”}:v_{i}=x_{r_{1}}+\;*\;\left(x_{r_{2}}-x_{r_{3}}\right), \end{array}$$

(5) $$\begin{array}{c} \text{“DE}/\text{best}/1\text{”}:v_{i}=x_{\text{best}}+F\;*\;\left(x_{r_{1}}-x_{r_{2}}\right), \end{array}$$

(6) $$\begin{array}{c} \text{“DE}/\text{current}‐\text{to}‐\text{best}/1\text{”}:v_{i}=x_{i}+F\;*\;\left(x_{\text{best}}-x_{i}\right)+F\;*\;\left(x_{r_{1}}-x_{r_{2}}\right), \end{array}$$

(7) $$\begin{array}{c} \text{“DE}/\text{best}/2\text{”}:v_{i}=x_{\text{best}}+F\;*\;\left(x_{r_{1}}-x_{r_{2}}\right)+F\;*\;\left(x_{r_{3}}-x_{r_{4}}\right), \end{array}$$

(8) $$\begin{array}{c} \text{“DE}/\mathrm{rand}/2\text{”}:v_{i}=x_{r_{1}}+F\;*\;\left(x_{r_{2}}-x_{r_{3}}\right)+F\;*\;\left(x_{r_{4}}-x_{r_{5}}\right), \end{array}$$

where *r*_1_, *r*_2_, *r*_3_, *r*_4_, *r*_5_ ∈ {1,…, *N*_*p*_} and *r*_1_ ≠ *r*_2_ ≠ *r*_3_ ≠ *r*_4_ ≠ *r*_5_ ≠ *i*. *v*_*i*_ is the mutant vector. *x*_best_ refers to the best fitness in the population at current generation. The scaling factor F controls the difference vectors with a positive value.

To diversify the current population, following mutation, DE uses a crossover operator to produce the trial vector *u*_*i*_ between *x*_*i*_ and *v*_*i*_. The most commonly used operator is the binomial crossover performed on each component as follows:

(9) $$\begin{array}{c} u_{i,j}=\left\{\begin{array}{cc} v_{i,j},\mathrm{rand}\left[0,1\right)\leq CR & \mathrm{or}\;j=j_{\mathrm{rand}},\\ x_{ij}, & \text{otherwise}, \end{array}\right. \end{array}$$

where *i*=1,…, *N*_*p*_, *j*=1,…, *D*, rand[0,1) represents a number drawn uniformly between 0 and 1, *CR* ∈ [0,1] is the crossover rate, and *j*_rand_ is a randomly selected integer within [1, *D*].

After mutation and crossover, each generated trial vector undergoes boundary constraint check. If some variables of trial vector are out of the boundary, a repair method is applied as follows:

(10) $$\begin{array}{c} x_{i,j}=x_{j}^{\min}+\mathrm{rand}\left[0,1\right]\cdot \left(x_{j}^{\max}-x_{j}^{\min}\right),if\;x_{i,j}<x_{j}^{\min}\;or\;{x\;}_{i,j}>x_{j}^{\max}, \end{array}$$

where *x*_*j*_^min^ and *x*_*j*_^max^ are the lower bound and upper bound, respectively, *x*_*j*_ ∈ [*x*_*j*_^min^, *x*_*j*_^max^]. rand[0,1] returns a uniformly distributed random number between 0 and 1 (0 ≤ rand[0,1] ≤ 1).

At last, the selection operator is performed to select the better one between *x*_*i*_ and *u*_*i*_ to enter the next generation:

(11) $$\begin{array}{c} x_{i}=\left\{\begin{array}{cc} u_{i}, & f\left(u_{i}\right)\leq f\left(x_{i}\right),\\ x_{i}, & \text{otherwise}, \end{array}\right. \end{array}$$

where *f*(*x*) is the objective function to be optimized.

## 4. Proposed Approach

In this section, we propose a novel DE, named HDEMR. HDEMR contains three main components: improved contraction criterion, BFGS local search, and model-based reinitialization strategy. And then, the complete proposed framework of HDEMR is shown in Algorithm 1. Next, the implementation of the above three main components will be introduced in detail.

### 4.1. Improved Contraction Criterion

In order to design an effective and efficient hybrid algorithm for global optimization, we need to take advantage of both the exploration capabilities of EA and the exploitation capabilities of LS and combine them in a well-balanced manner. For successful incorporation of LS in DE, a triggering condition, called contraction criterion, is needed to decide when the local search has to start. There are several kinds of methods to define a contraction criterion. Qin and Suganthan [48] applied a local search method after a fixed number of generations (every 200 generations). Sun et al. [49] used the condition that if the promising solution was not updated in *t*-consecutive generations, LS would start. Simon et al. [50] used the minimum fitness in the objective space as the contraction criterion. Vasile et al. [23] performed LS when the maximum distance in decision space was below a given threshold. Peng et al. [43] proposed a new contraction criterion which combined the improved maximum distance in objective space and the maximum distance in decision space.

To have a good judgement to the individuals' distribution, a good measure to evaluate the crowding degree around each solution is needed as a mixed triggering condition. In HDEMR, we also propose an improved contraction criterion which combines two criteria: (a) *E* is the population entropy in objective space and (b) *S* is the maximum distance in decision space.

**Theorem 1 .**

Let R be the range of the fitness function values of the population in the proposed algorithm. If *A*_1_ ∪ *A*_2_ ∪ … ∪ *A*_*n*_=*R* and *A*_1_ ∩ *A*_2_ ∩ … ∩ *A*_*n*_=∅, where *A*_1_, *A*_2_,….,*A*_*n*_ are the true subsets of the population, the population entropy of HDEMR is defined as:

(12) $$\begin{array}{c} E=-\overset{n}{\underset{i=1}{\sum }}\left(p_{i}\cdot \log\;p_{i}\right),{\;p}_{i}=s_{i}/N_{p}, \end{array}$$

where *s*_*i*_ is the number of individuals in the subset *A*_*i*_ and *N*_*p*_ is the population size in HDEMR. *E* is the measure to represent the diversity of the population in objective space.

The distance in decision space is defined as

(13) $$\begin{array}{c} S=\text{Max}\left(\left\|x_{i}-x_{j}\right\|\right),\;\forall x_{i},x_{j}\in P, \end{array}$$

where ‖·‖ is the Euclidean distance and *S* is the measure to represent the diversity of the population in decision space.

### 4.2. BFGS Local Search

The local search utilizes the better solutions obtained by the global search to update the population and thus enhances algorithm's exploitation ability to find the best solution. In HDEMR, we use the BFGS algorithm as the local search method. It is one of the quasi-Newton methods which do not need the precise Hessian matrix and be able to approximate it based on the individual successive gradients. BFGS is considered to be the most effective and popular quasi-Newton method and has been proven to have good performance for solving unconstrained nonlinear optimization problems. The details can be found in [51, 52].

### 4.3. Model-Based Reinitialization Strategy

After the local search is performed, if the best solution has not been improved, a reinitialization of the whole population is used to give the algorithms more opportunities to find the global optimum. Sun et al. [49] chose the individuals that had the largest distances from the local optima to form the next population from a temporary population. Simon et al. [50] proposed a partial reinitialization of the population. Every 20 generations, the algorithm selected the best *M* individuals from a temporary population of 2*M*+2 individuals as the reinitialization pool. Zamuda et al. [53] proposed a population size reduction method as the reinitialization strategy. In this paper, we propose a model-based reinitialization strategy. If the local search does not improve the best solution in the current generation, the population will be restarted. A counter *C* keeps track of the number of restarts. For *C* < *C*_max_, where *C*_max_ is user-defined, the new population, *ℙ*′, is generated randomly in the search space. For *C* ≥ *C*_max_, *ℙ*′ is initialised from a Gaussian distribution model base on the mean and standard deviation of the best solutions in the Archive.  Algorithm 2 summarises the model-based reinitialization procedure.

## 5. Experimental Study

To assess the performance of HDEMR, three groups of experiments are conducted. We compare HDEMR with seven well-known algorithms including JADE [54], CoDE [55], jDE [56], MPEDE [57], SHADE [58], LSHADE [44], and LSHADE-*ε* [59]. In the first group of experiments, we select the 21 nonnoisy benchmark functions (excluding noisy functions F4, F17, F24, and F25) at IEEE CEC2005 [60]. In the second group of experiments, we use 30 test functions at IEEE CEC2014 [61] to further demonstrate the effectiveness of HDEMR. In the third group of experiments, we compare HDEMR with the well-known and widely used deterministic algorithm DIRECT [15, 16] on GKLS test classes [62] to further verify the performance of HDEMR.

### 5.1. Performance Test of HDEMR on CEC2005 Benchmark Functions

For each algorithm, we conduct 25 independent runs and limit each run to 10000∗*D* max function evaluations at 21 benchmark problems as in [60]. Because the dimension of weight parameter identification of ILAIS is approximately 10, HDEMR is just tested by the benchmark problems at *D*=10 to evaluate its performance at low-dimensional problem. The algorithms are evaluated in terms of function error value [60], defined as *f*(*x*) − *f*(*x*^*∗*^), where *x*^*∗*^ is the global optimum of the test function. Mean error and standard deviation of the function error values are recorded. The parameters of HDEMR are set as *N*_*p*_=30, *E*_max_=3.0, *S*_max_=2.0, *C*_max_=9, *CR* ∈ *N*(0.8, 0.1), and *F* ∈ *N*(0.5, 0.1). For the other seven well-known algorithms, we use the same parameter settings as in their original papers.

To effectively analyze the results, two nonparametric statistical tests, as similarly done in [63], are used in the experiments. (i) The Wilcoxon rank-sum test at *α*=0.05 is performed to test the statistical significance of the experimental results between two algorithms in single-problem and multiproblem. (ii) The Friedman test is employed to obtain the average rankings of all the compared algorithms. The Wilcoxon rank-sum test in single-problem is calculated by Matlab, while the Wilcoxon test in multiproblem and the Friedman test are carried out by the software KEEL [64].

According to the results shown in Table 2 (last three rows), HDEMR performs significantly better than JADE on 8 test functions, and JADE wins in 5 test functions. Compared with CoDE, HDEMR shows better and worse performance on 10 test functions and 7 test functions, respectively. HDEMR wins 12 test functions and loses 3 test functions to jDE. MPEDE surpasses HDEMR on 2 test functions; however, HDEMR is significantly better than MPEDE on 11 test functions. HDEMR outperforms SHADE on 5 test functions and is worse than SHADE on 7 test functions. Moreover, HDEMR performs better than LSHADE on 3 functions and worse than LSHADE on 8 functions. Compared with LSHADE-*ε*, HDEMR shows better and worse performance on 8 test functions and 7 test functions, respectively. Furthermore, HDEMR obtains similar results with other seven algorithms in 8, 4, 6, 8, 9, 10, and 6 cases.

In addition, some interesting phenomena can be observed according to the experimental results in Table 2. For the unimodal functions (i.e., F1, F2, and F3), the performance of HDEMR is significantly better than that of the other algorithms. Moreover, HDEMR also outperforms the other algorithms on F6 and F8. To hybrid composition functions (i.e., F18–F23), HDEMR exhibits the best performance among the eight algorithms. The outstanding performance of HDEMR on hybrid composition functions can be attributed to its capability to balance the exploration and exploitation. JADE, CoDE, SHADE, LSHADE, and LSHADE-ε perform quite well on the expanded multimodal test functions (i.e., F13 and F14), which means history-based parameter and trial vector generation adaptation strategies can effectively improve the performance of advance DE to the expanded multimodal functions.

In order to further validate the performance of model-based reinitialization strategy, we compare HDEMR with HDE (hybrid differential evolution with reinitialization randomly).  Table 3 shows the results of HDEMR and HDE on CEC2005 benchmark functions. It can be seen that HDEMR performs significantly better than HDE on 8 test functions. HDE wins on 1 test function. Also, HDEMR obtains similar results with HDE on 12 test functions. Some interesting phenomena can be observed according to the experimental results. HDEMR performs quite well on most of the hybrid composition functions (i.e., F15 and F18–F22), which further demonstrates that model-based reinitialization strategy can effectively improve the performance of HDEMR to the difficult functions.

**Figure 1:**
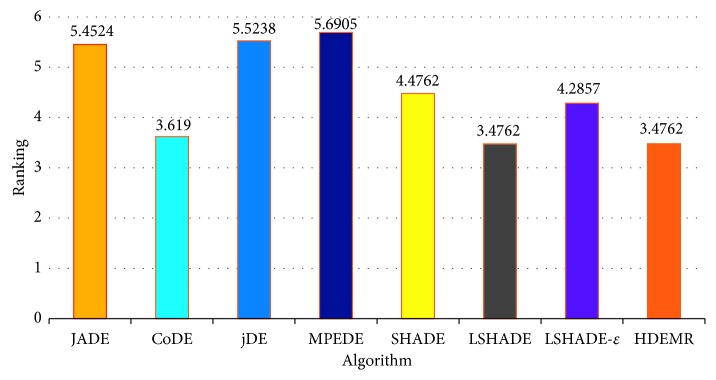
Average rankings of the eight algorithms by Friedman test for CEC2005 at *D*=10.

To further detect the significant differences between HDEMR and the seven competitors, the multiple-problem Wilcoxon test and the Friedman test are carried out, respectively. As shown in Table 4, it can be seen that HDEMR can obtain higher *R*^+^ values than *R*^−^ values in all cases, where *R*^+^ is the sum of ranks for the functions on which HDEMR outperforms the compared algorithm, and *R*^−^ is the sum of ranks for the opposite [63]. According to the Wilcoxon test at *α*=0.05, the significant differences can be observed in three cases (i.e., HDEMR vs. JADE, HDEMR vs. jDE, and HDEMR vs. MPEDE). When *α*=0.1, there are significant differences in five cases (i.e, HDEMR vs. JADE, HDEMR vs. jDE, HDEMR vs. MPEDE, HDEMR vs. SHADE, and HDEMR vs. LSHADE-*ε*), which means that HDEMR is significantly better than JADE, jDE, MPEDE, SHADE, and LSHADE-*ε* on 21 test functions at *α*=0.1. Moreover, the results shown in Figure 1 indicate that HDEMR and LSHADE have the best ranking (3.4762) among the eight compared algorithms. In general, the above comparison clearly demonstrates that HDEMR and LSHADE are significantly better than the other competitors at CEC2005 benchmark functions.

### 5.2. Sensitivity in Relation to the Parameters *E*_max_ and *S*_max_

The improved contraction criterion in HDEMR contains two parameters *E*_max_ and *S*_max_. They are the triggering condition to the local search. In order to investigate the sensitivity of the above two parameters, we test HDEMR with different *E*_max_={1.0, 2.0, 3.0} and *s*_max_={1.0, 2.0, 3.0}. Nine different combinations of *E*_max_ and *S*_max_ are done. The statistical results by the Friedman test with all initial values are shown in Table 5.

From Table 5, we can see that HDEMR provides the best average ranking value at *E*_max_=3.0 and *S*_max_=2.0 than other 8 combinations on the test functions. In general, we can conclude that it is better to set a large value to *E*_max_ and a small value to *S*_max_.

### 5.3. Sensitivity in Relation to the Parameter *C*_max_

In order to test the influence of the parameter *C*_max_ used in the reinitialization scheme of HDEMR, a set of experiments is performed here. The Friedman test results are shown in Table 6, where the values of *C*_max_ are set to *C*_max_={3,5,7,9,11,13,15}. All other parameters are kept unchanged as described in Section 5. In addition, all experiments are conducted for 25 independent runs for each function.

It can be seen from Table 6 that HDEMR with *C*_max_=9 gets better average ranking value (3.3333) than other six cases. *C*_max_=15 is the second best choice to HDEMR. Some interesting phenomena can be observed in Table 6. The large *C*_max_ (i.e., 13 and 15) is better than the small values (i.e., 3 and 5). Generally speaking, the large *C*_max_ value such as 9 or 15 is good to enhance the performance of HDEMR.

### 5.4. Performance Test of HDEMR on CEC2014 Benchmark Functions

**Figure 2:**
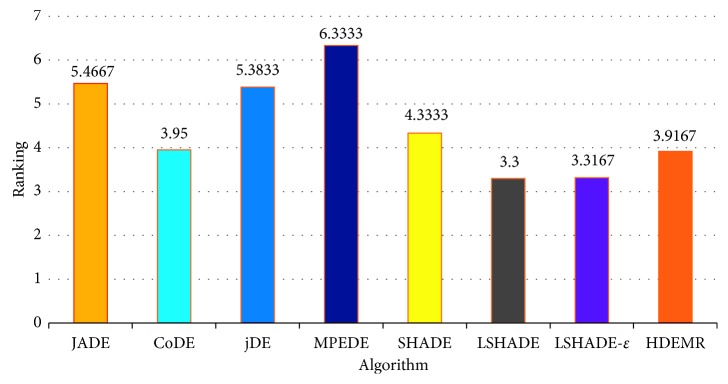
Average rankings of the eight algorithms by Friedman test for CEC2014 at *D*=10.

**Figure 3:**
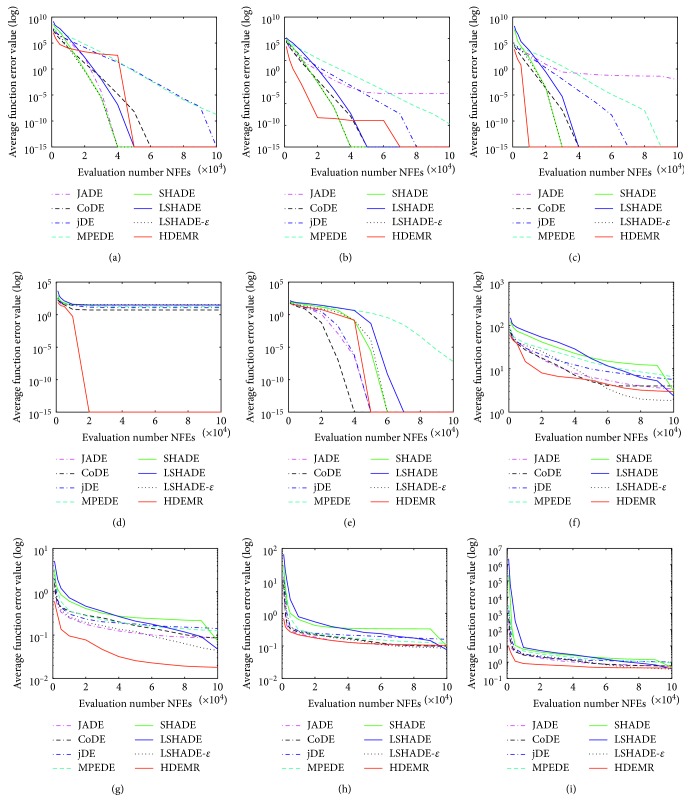
The convergence graphs on different types of CEC2014 benchmark functions. (a) The convergence graph on the unimodal function f1. (b) The convergence graph on the unimodal function f2. (c) The convergence graph on the unimodal function f3. (d) The convergence graph on the simple multimodal function f4. (e) The convergence graph on the simple multimodal function f8. (f) The convergence graph on the simple multimodal function f9. (g) The convergence graph on the simple multimodal function f13. (h) The convergence graph on the simple multimodal function f14. (i) The convergence graph on the simple multimodal function f15.

**Figure 4:**
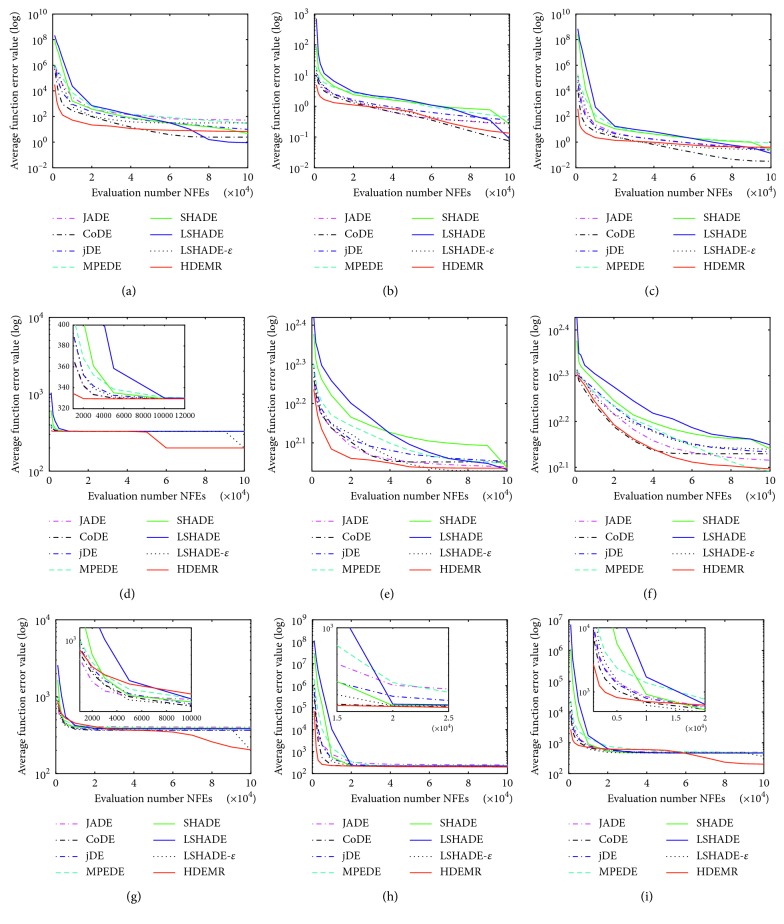
The convergence graphs on different types of CEC2014 benchmark functions. (a) The convergence graph on the hybrid function f17. (b) The convergence graph on the hybrid function f19. (c) The convergence graph on the hybrid function f20. (d) The convergence graph on the composition function f23. (e) The convergence graph on the composition function f24. (f) The convergence graph on the composition function f25. (g) The convergence graph on the composition function f28. (h) The convergence graph on the composition function f29. (i) The convergence graph on the composition function f30.

In this section, we compare our approach with the same seven algorithms at 30 benchmark problems of CEC2014 as in [61]. All functions are conducted for 51 independent runs and limit each run to 10000∗*D* max function evaluations. The average function error value smaller than 10^−8^ is taken as zero. The parameters of HDEMR are set as *N*_*p*_=30, *E*_max_=3.0, *S*_max_=2.0, *C*_max_=9, *CR* ∈ *N*(0.8, 0.1), and *F* ∈ *N*(0.5, 0.1). For the other seven well-known algorithms, we use the same parameter settings as in their original papers.  Table 7 shows mean error and standard deviation of the function error values.  Table 8 shows the multiple-problem Wilcoxon test results.  Figure 2 is the Friedman test results. Additionally, some representative convergence graphs of eight algorithms are shown in Figures 3 and 4.

From Table 7, we can see that HDEMR performs better than all of the algorithms except LSHADE and LSHADE-*ε* at CEC2014 functions. Among the 30 test functions, HDEMR is significantly better than JADE, CoDE, jDE, MPEDE, SHADE, LSHADE, and LSHADE-*ε* in 16, 13, 19, 23, 12, 9, and 7 functions, respectively, while the number of functions that JADE, CoDE, jDE, MPEDE, SHADE, LSHADE, and LSHADE-*ε* performs significantly better are 4, 10, 3, 2, 7, 15, and 11. Furthermore, HDEMR obtains similar results with other seven algorithms in 10, 7, 8, 5, 11, 6, and 12 cases. From Table 8, we can find that there are the significant differences in three cases (i.e., HDEMR vs. JADE, HDEMR vs. jDE, and HDEMR vs. MPEDE) at *α*=0.05 and *α*=0.1. Moreover, the results shown in Figure 2 indicate that HDEMR ranks the third best (3.9167) among the eight compared algorithms.

In addition, some interesting phenomena can be observed according to the experimental results in Table 7. For the unimodal functions (i.e., F1, F2, and F3), HDEMR can produce similar results compared with the other algorithms. To simple multimodal functions and hybrid functions, HDEMR has better or similar results than JADE, CoDE, jDE, and MPEDE. LSHADE and LSHADE-*ε* are very good at solving the simple multimodal and hybrid functions. To the composition functions (i.e., F23–F30), HDEMR exhibits the best performance among the eight algorithms. The outstanding performance of HDEMR on the composition functions can further demonstrate its capability to balance the exploration and exploitation. Some representative convergence graphs of the eight algorithms in some typical test functions have been shown in Figures 3 and 4. Figures 3 and 4 show that HDEMR has a fast convergence speed at most of the functions, especially on the composition functions at CEC2014 benchmark.

### 5.5. Performance Test of HDEMR on GKLS Test Classes

To further validate the performance of HDEMR, the popular GKLS generator is used. This generator allows one to randomly generate classes of test problems with the same dimension, number of local minima, and difficulty, and each class contains 100 problems. Here, 1000 test functions are used in total, which consist of 10 classes of problems with dimension *D* = 2, 3, 4, 5, and 10. The control parameters of GKLS test classes used in the experiments are presented in Table 9. Control parameters of the GKLS generator include (1) the radius of the convergence region *ρ*, (2) distance between the paraboloid vertex and the global minimizer *r*, and (3) the tolerance Δ (for details, see Sergeyev and Kvasov [14]). The parameters of HDEMR are set as *N*_*p*_=30, *E*_max_=3.0, *S*_max_=2.0, *C*_max_=9, *CR* ∈ *N*(0.8, 0.1), and *F* ∈ *N*(0.5, 0.1). In order to make this comparison more reliable, parameters of DIRECT are used as recommended in its original papers. For each of the generated problems, HDEMR is conducted separately 100 independent runs and DIRECT is executed one run. The maximal number of trails (or function evaluations) is Max_NFEs=10^6^.

To effectively analyze the results, Operational Characteristics and Operational Zones, as similarly done in [13], are used in the experiments.

**Figure 5:**
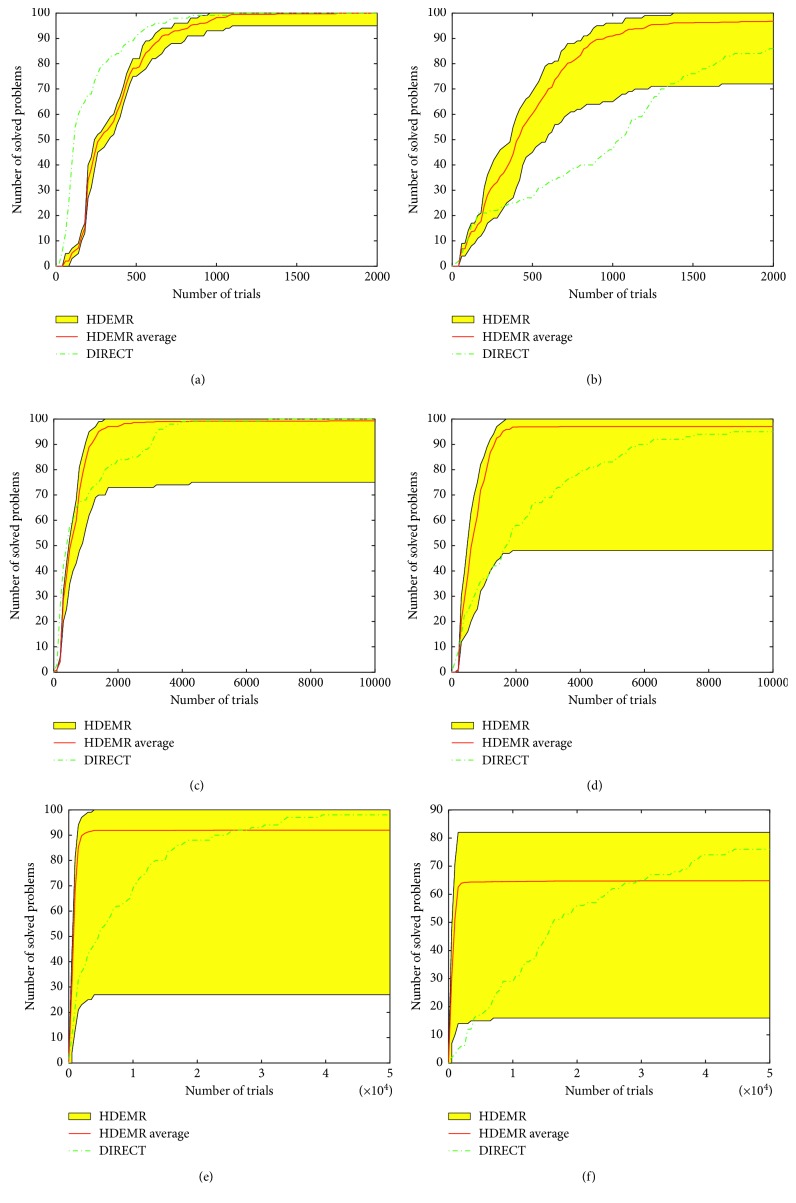
Operational characteristics built on 2-, 3-, 4-dimensional classes of GKLS test functions for DIRECT and operational zones for HDEMR. (a) Operational characteristics for DIRECT and the operational zone for HDEMR for the simple 2-dimensional class. (b) The same as (a) for the hard class. (c) Operational characteristics for DIRECT and the operational zone for HDEMR for the simple 3-dimensional class. (d) The same as (c) for the hard class. (e) Operational characteristics for DIRECT and the operational zone for HDEMR for the simple 4-dimensional class. (f) The same as (e) for the hard class.

**Figure 6:**
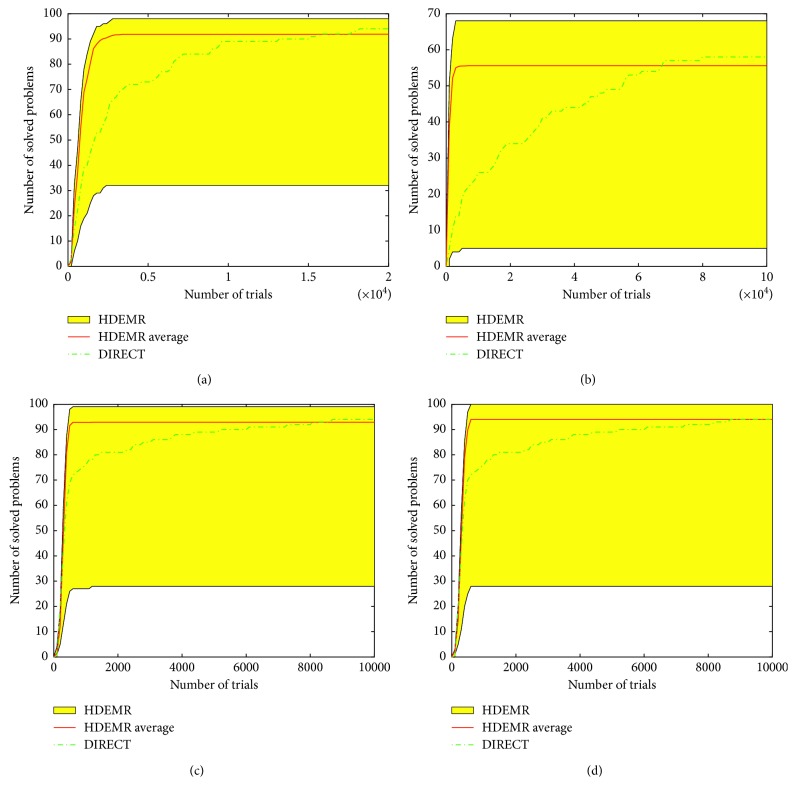
Operational characteristics built on 5- and 10-dimensional classes of GKLS test functions for DIRECT and operational zones for HDEMR. (a) Operational characteristics for DIRECT and the operational zone for HDEMR for the simple 5-dimensional class. (b) The same as (a) for the hard class. (c) Operational characteristics for DIRECT and the operational zone for HDEMR for the simple 10-dimensional class. (d) The same as (c) for the hard class.

Because of the huge amount of data, only average results are included in Table 10. *m*(*i*) means that the algorithm does not solve a global optimization problem *i* times in 100 runs × 100 problems (i.e., in 10,000 runs for HDEMR and in 100 runs for DIRECT). Figures 5 and 6 show operational characteristics for DIRECT and operational zones for HDEMR on the different dimensions.

Figure 5 shows the results on the 2-, 3-, 4-dimensional simple and hard classes for HDEMR and DIRECT. It can be seen from Figure 5(a) that operational characteristics of DIRECT is higher than the upper boundary of the zone of HDEMR and, therefore, on this class DIRECT has a better performance.  Figure 5(b) shows that the average performance with the zone (the red line in the zone) of HDEMR is higher than characteristics of DIRECT. It means that HDEMR outperforms DIRECT. Figures 5(c) and 5(d) show that the average performance of HDEMR is better than DIRECT on both the 3-dimensional simple and hard classes.

Figures 5(e) and 5(f) depict that on the 4-dimensional simple and hard classes, if the number of function evaluations is less than 30000, the average performance of HDEMR is better than DIRECT. If the number of function evaluations is higher than 30000, DIRECT behaves better since its characteristic is higher than the average line of the HDEMR zone. We can also see the similar results on the 5-dimensional simple and hard classes in Figures 6(a) and 6(b).

Figures 6(c) and 6(d) illustrate that the average performance of HDEMR is better than DIRECT on the 10-dimensional simple and hard classes. It is suitable to solve the ILAIS problem because the dimension of weight parameter identification of ILAIS is approximately 10.

In addition, some interesting phenomena can be observed according to the experimental results in Figures 5 and 6. The red average lines indicate that HDEMR has a fast convergence speed at most of the classes except for 2-dimensional simple class, especially on the hard classes. However, the number of solved problems by DIRECT is more than HDEMR on 4-dimensional and 5-dimensional hard classes (see Figures 5(f) and 6(b)).

At last, one can see that the performance of HDEMR is not very stable on 4-, 5-, 10-dimensional classes. The reason is that HDEMR gets trapped into local minima in several cases of 100 runs and is not able to exit from their attraction regions producing the operational zones with the wider interval.

From Table 10, the number of solved problems of DIRECT is more than HDEMR at GKLS test classes. For example, on the 10-dimensional hard class, DIRECT does not solve 2 problems in 100 runs (98% of success), whereas HDEMR does not solve 599 problems in 10000 runs (94.01% of success). On the other hand, the average number of trails (or function evaluations) for DIRECT is 21774.72, while for HDEMR is 60191.

## 6. HDEMR for Weight Parameter Optimization of ILAIS

According to the results of CEC2005, CEC2014, and GKLS test functions, HDEMR achieves better or similar results among nine algorithms. Hence, it is a good alternative for weight parameter optimization of ILAIS. In this section, the experiments aim at using HDEMR to solve the weight parameter identification of ILAIS.

Questionnaire survey and individual evaluation are conducted at China University of Geosciences. There are 1000 questionnaires collected from the students, and 300 questionnaires from the teachers.

The parameters of HDEMR are set as *N*_*p*_=100, *E*_max_=3.0, *S*_max_=2.0, *C*_max_=9, *CR* ∈ *N*(0.8, 0.1), *F* ∈ *N*(0.5, 0.1), and Max_NFEs=50000. HDEMR is conducted for 50 independent runs. The optimization objective function is defined in Equation (2) and *t*=0.6, *s*=0.4. To effectively analyze the results, mean value and standard deviation are also used in the experiments.

Table 11 shows the weight results of the information literacy assessment index at CUG by HDEMR. It can be seen that the weights of first-level indexes such as Information Consciousness, Information Knowledge, Information Ability, and Information Morality are 0.1958, 0.2085, 0.3115, and 0.2958, respectively. Information Ability and Information Morality are more important than Information Consciousness and Information Knowledge according to the questionnaire survey. It means that Information Ability and Information Morality have a greater impact to assess the information literacy of the college students.

Table 11 also shows the results of the thirteen second-level index weights by HDEMR. We can see that the weight of L11 is 0.4031 and L12 and L13 are approximately 0.3 which means that L11 (the recognition of the value of information and the objective evaluation of the role of information) is an important weight to Information Consciousness. To Information Knowledge, the results present that the weights of L22 and L23 are both approximately 0.4 which means that the ability of understanding the value of various sources of information and communication process and the abilities of identifying information needs, mastering basic information science knowledge, clarifying your own information needs, and describing keywords and terminology are very important to assess the collage students' Information Knowledge. Furthermore, it is also seen that the weights of L32, L33, and L34 are close to 0.3. It means that these three indexes have the same effect on Information Ability. At last, to Information Morality, the weight of L41 is close to 0.5. This means that the ability to master information law has the significant impact on evaluating the college students' Information Morality. The second important factor of L42 is 0.2983 to Information Morality.

Finally, according to the results in Table 11, it can be seen that all the standard deviations are within 10^−3^. It can draw the conclusion that HDEMR is stable to optimize the weights of ILAIS. The optimal weights are able to objectively determine the evaluation of the college students' information literacy at CUG.

## 7. Conclusion

Apart from developing a new weight parameter optimization model of ILAIS, in this study, an efficient HDEMR method is proposed to solve the weight parameter identification problem of ILAIS at CUG with greater speed and accuracy. The HDEMR method uses an improved contraction criterion to decide when the local search starts. A model-based reinitialization strategy is also proposed to improve the diversity of the algorithm and the performance of global search. These improvements are simple yet efficient and make HDEMR a powerful alternative for the real-world applications. The effectiveness and efficiency of HDEMR are validated by CEC2005, CEC2014 benchmark functions, and GKLS test classes. The superiority of HDEMR is also verified after comparing it with seven well-known DE variants and the widely used deterministic algorithm DIRECT. At last, HDEMR is successfully used to optimize the weight parameters of ILAIS at China University of Geosciences (CUG).

In our future work, HDEMR will be tested on other real-world applications problems. Moreover, we believe that some other local search algorithms and parameter adaptation strategies can also be used in HDEMR.

## Figures and Tables

**Algorithm 1:**
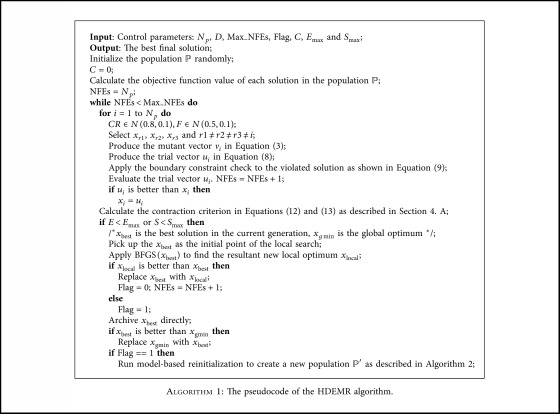
The pseudocode of the HDEMR algorithm.

**Algorithm 2:**
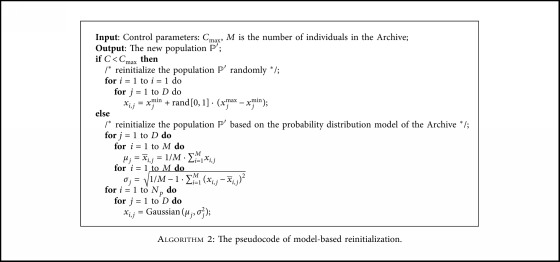
The pseudocode of model-based reinitialization.

**Table 1:** Information literacy assessment index system at CUG.

First-level index	Second-level index
L1: Information Consciousness	L11: the recognition of the value of information and the objective evaluation of the role of information
	L12: attitudes towards various social problems involving in the process of access to and use of information
	L13: recognise the right useful information

L2: Information Knowledge	L21: effectively select information retrieval tools and know the advantages and disadvantages of different information retrieval tools
	L22: have information source knowledge, and understand the value of various sources of information and communication process
	L23: identify reliable and significant information sources, master basic information science knowledge

L3: Information Ability	L31: the ability of information retrieval
	L32: the ability to use and process information
	L33: the ability to share, deliver, and create information ability
	L34: the ability to learn information knowledge independently and information development cooperation

L4: Information Morality	L41: ability to master information law
	L42: information security and privacy
	L43: information ethic cognition and behavior

**Table 2:** Comparison of mean error and standard deviation between HDEMR and other seven DE variants on CEC2005 at *D*=10.

Function	JADE			CoDE			jDE			MPEDE			SHADE			LSHADE			LSHADE-ε			HDEMR	
F01^*∗*^	6.27*E* – 03	3.07*E* – 03	–	1.07*E* – 02	7.14*E* – 03	–	1.29*E* – 01	5.39*E* – 02	–	2.32*E* + 01	6.00*E* + 00	–	2.39*E* – 02	1.38*E* – 02	–	6.66*E* – 08	1.05*E* – 07	–	1.58*E* – 06	1.18*E* – 06	–	0.00*E* + 00	0.00*E* + 00
F02	0.00*E*+00	0.00*E*+00	=	3.03*E* – 30	1.51*E* – 29	=	2.74*E* – 17	6.07*E* – 17	–	3.46*E* – 15	8.73*E* – 15	–	0.00*E* + 00	0.00*E* + 00	=	0.00*E* + 00	0.00*E* + 00	=	0.00*E* + 00	0.00*E* + 00	=	0.00*E* + 00	0.00*E* + 00
F03	4.81*E* – 26	1.80*E* – 26	–	5.02*E* – 16	1.39*E* – 15	–	1.54*E* – 06	6.01*E* – 06	–	8.61*E* – 11	1.67*E* – 10	–	8.45*E* – 26	2.45*E* – 26	–	0.00*E* + 00	0.00*E* + 00	=	5.96*E* – 26	2.70*E* – 26	–	0.00*E* + 00	0.00*E* + 00
F05	0.00*E* + 00	0.00*E* + 00	+	0.00*E* + 00	0.00*E* + 00	+	7.28*E* – 14	3.64*E* – 13	+	2.99*E* – 06	1.70*E* – 06	+	0.00*E* + 00	0.00*E* + 00	+	0.00*E* + 00	0.00*E* + 00	+	0.00*E* + 00	0.00*E* + 00	+	1.53*E* – 02	1.09*E* – 02
F06	2.35*E* + 00	2.56*E* + 00	–	1.02*E* – 23	3.42*E* – 23	–	3.97*E* – 02	7.00*E* – 02	–	1.16*E* – 01	9.43*E* – 02	–	0.00*E* + 00	0.00*E* + 00	=	0.00*E* + 00	0.00*E* + 00	=	0.00*E* + 00	0.00*E* + 00	=	0.00*E* + 00	0.00*E* + 00
F07	2.12*E* – 02	9.98*E* – 03	=	5.06*E* – 02	4.16*E* – 02	–	1.27*E* + 03	4.20*E* – 13	–	2.07*E* – 02	2.03*E* – 02	=	5.75*E* – 03	8.77*E* – 03	+	3.84*E* – 03	6.20*E* – 03	+	2.85*E* – 03	4.88*E* – 03	+	1.93*E* – 02	1.31*E* – 02
F08	2.03*E* + 01	6.99*E* – 02	–	2.01*E* + 01	1.15*E* – 01	–	2.03*E* + 01	7.47*E* – 02	–	2.03*E* + 01	8.21*E* – 02	–	2.02*E* + 01	1.60*E* – 01	–	2.01*E* + 01	1.03*E* – 01	–	2.01*E* + 01	1.09*E* – 01	–	2.00*E* + 01	0.00*E* + 00
F09^*∗*^	1.33*E* + 01	1.88*E* + 00	–	7.47*E* + 00	1.97*E* + 00	–	9.78*E* + 00	2.07*E* + 00	–	2.64*E* + 01	4.23*E* + 00	–	1.79*E* + 01	2.76*E* + 00	–	8.37*E* – 01	7.90*E* – 01	+	7.79*E* + 00	1.82*E* + 00	–	4.57*E* + 00	5.13*E* – 01
F10	4.21*E* + 00	1.01*E* + 00	+	7.96*E* + 00	2.72*E* + 00	–	1.04*E* + 01	2.75*E* + 00	–	6.92*E* + 00	1.95*E* + 00	–	2.73*E* + 00	9.31*E* – 01	+	2.47*E* + 00	1.04*E* + 00	+	1.75*E* + 00	8.27*E* – 01	+	5.77*E* + 00	1.57*E* + 00
F11	4.40*E* + 00	8.05*E* – 01	+	4.87*E* – 01	6.39*E* – 01	+	5.86*E* + 00	6.98*E* – 01	=	5.68*E* + 00	6.59*E* – 01	=	4.88*E* + 00	7.14*E* – 01	+	4.12*E* + 00	7.50*E* – 01	+	4.43*E* + 00	6.47*E* – 01	+	6.06*E* + 00	8.05*E* – 01
F12	1.41*E* + 01	1.98*E* + 01	–	1.16*E* + 01	4.24*E* + 01	–	6.55*E* + 01	3.11*E* + 02	–	2.32*E* + 00	5.68*E* + 00	–	3.05*E* + 01	1.42*E* + 02	–	2.40*E* + 00	4.36*E* + 00	–	3.60*E* + 00	4.90*E* + 00	–	0.00*E* + 00	0.00*E* + 00
F13	3.39*E* – 01	3.80*E* – 02	=	2.91*E* – 01	1.07*E* – 01	+	4.35*E* – 01	5.87*E* – 02	=	5.30*E* – 01	6.54*E* – 02	–	2.82*E* – 01	4.65*E* – 02	+	2.31*E* – 01	3.67*E* – 02	+	2.21*E* – 01	4.24*E* – 02	+	3.96*E* – 01	1.12*E* – 01
F14	2.75*E* + 00	2.47*E* – 01	+	2.17*E* + 00	7.65*E* – 01	+	3.36*E* + 00	1.63*E* – 01	–	3.10*E* + 00	2.21*E* – 01	=	2.58*E* + 00	3.38*E* – 01	+	2.40*E* + 00	2.93*E* – 01	+	2.09*E* + 00	3.73*E* – 01	+	3.12*E* + 00	3.08*E* – 01
F15	9.38*E* + 01	1.47*E* + 02	=	8.01*E* + 01	1.44*E* + 02	=	1.65*E* + 01	3.08*E* + 01	+	2.86*E* + 01	8.22*E* + 01	=	1.05*E* + 02	1.72*E* + 02	=	1.78*E* + 02	1.90*E* + 02	=	1.07*E* + 02	1.57*E* + 02	=	3.55*E* + 01	3.13*E* + 01
F16	9.76*E* + 01	3.34*E* + 00	+	1.02*E* + 02	9.54*E* + 00	+	1.13*E* + 02	7.42*E* + 00	=	1.02*E* + 02	5.51*E* + 00	+	9.39*E* + 01	6.78*E* + 00	+	9.26*E* + 01	2.66*E* + 00	+	9.13*E* + 01	5.07*E* + 00	+	1.11*E* + 02	8.68*E* + 00
F18	6.33*E* + 02	2.56*E* + 02	=	5.00*E* + 02	2.50*E* + 02	+	5.60*E* + 02	2.55*E* + 02	=	6.00*E* + 02	2.50*E* + 02	=	6.03*E* + 02	2.53*E* + 02	=	6.00*E* + 02	2.50*E* + 02	=	6.70*E* + 02	2.21*E* + 02	=	5.15*E* + 02	2.23*E* + 02
F19	6.75*E* + 02	2.40*E* + 02	–	5.00*E* + 02	2.50*E* + 02	=	4.84*E* + 02	2.51*E* + 02	=	6.80*E* + 02	2.18*E* + 02	–	5.64*E* + 02	2.59*E* + 02	=	6.40*E* + 02	2.38*E* + 02	=	7.02*E* + 02	2.05*E* + 02	–	4.51*E* + 02	2.06*E* + 02
F20	6.42*E* + 02	2.40*E* + 02	=	4.40*E* + 02	2.29*E* + 02	+	4.20*E* + 02	2.18*E* + 02	+	6.40*E* + 02	2.38*E* + 02	=	5.63*E* + 02	2.59*E* + 02	=	6.20*E* + 02	2.45*E* + 02	=	7.20*E* + 02	1.87*E* + 02	=	5.17*E* + 02	2.38*E* + 02
F21	5.12*E* + 02	2.03*E* + 02	–	4.98*E* + 02	9.05*E* + 01	–	5.28*E* + 02	1.10*E* + 02	–	4.60*E* + 02	1.38*E* + 02	=	4.84*E* + 02	1.68*E* + 02	=	4.88*E* + 02	1.90*E* + 02	=	5.84*E* + 02	2.10*E* + 02	–	3.70*E* + 02	1.31*E* + 02
F22	7.51*E* + 02	1.74*E* + 01	=	7.15*E* + 02	8.69*E* + 01	=	7.43*E* + 02	9.24*E* + 01	–	7.61*E* + 02	4.41*E* + 00	–	7.48*E* + 02	1.28*E* + 01	=	7.45*E* + 02	1.20*E* + 01	=	7.48*E* + 02	1.65*E* + 01	=	6.01*E* + 02	2.16*E* + 02
F23	6.64*E* + 02	1.22*E* + 02	=	5.89*E* + 02	9.12*E* + 01	–	6.28*E* + 02	1.40*E* + 02	=	6.18*E* + 02	7.92*E* + 01	=	6.38*E* + 02	1.24*E* + 02	=	6.44*E* + 02	1.24*E* + 02	=	7.40*E* + 02	1.86*E* + 02	–	5.60*E* + 02	6.57*E* – 01
+			5			7			3			2			7			8			7		
–			8			10			12			11			5			3			8		
=			8			4			6			8			9			10			6		

^*∗*^When seven algorithms obtain the global optimum, the intermediate results are reported at NFEs=10000. “+,” “–,” and “=” denote that the performance of this algorithm is, respectively, better than, worse than, and similar to HDEMR according to the Wilcoxon rank-sum test at *α*=0.05.

**Table 3:** Comparison of mean error and standard deviation between HDEMR and HDE over 25 independent runs on twenty-one 10-dimensional test functions.

Function	HDE			HDEMR	
F01^*∗*^	0.00*E* + 00	0.00*E* + 00	=	0.00*E* + 00	0.00*E* + 00
F02	2.27*E* – 15	1.14*E* – 14	=	0.00*E* + 00	0.00*E* + 00
F03	0.00*E* + 00	0.00*E* + 00	=	0.00*E* + 00	0.00*E* + 00
F05	1.79*E* – 02	1.32*E* – 02	–	1.53*E* – 02	1.09*E* – 02
F06	0.00*E* + 00	0.00*E* + 00	=	0.00*E* + 00	0.00*E* + 00
F07	2.02*E* – 02	1.00*E* – 02	=	1.93*E* – 02	1.31*E* – 02
F08	2.00*E* + 01	0.00*E* + 00	=	2.00*E* + 01	0.00*E* + 00
F09^*∗*^	3.13*E* + 00	8.03*E* – 01	+	4.57*E* + 00	5.13*E* – 01
F10	5.69*E* + 00	1.51*E* + 00	=	5.77*E* + 00	1.57*E* + 00
F11	6.06*E* + 00	1.12*E* + 00	=	6.06*E* + 00	8.05*E* – 01
F12	0.00*E* + 00	0.00*E* + 00	=	0.00*E* + 00	0.00*E* + 00
F13	4.32*E* – 01	1.01*E* – 01	–	3.96*E* – 01	1.12*E* – 01
F14	3.09*E* + 00	2.85*E* – 01	=	3.12*E* + 00	3.08*E* – 01
F15	4.26*E* + 01	3.74*E* + 01	–	3.55*E* + 01	3.13*E* + 01
F16	1.10*E* + 02	9.80*E* + 00	=	1.11*E* + 02	8.68*E* + 00
F18	5.59*E* + 02	2.38*E* + 02	–	5.15*E* + 02	2.23*E* + 02
F19	6.08*E* + 02	2.24*E* + 02	–	4.51*E* + 02	2.06*E* + 02
F20	6.07*E* + 02	2.27*E* + 02	–	5.17*E* + 02	2.38*E* + 02
F21	4.08*E* + 02	1.29*E* + 02	–	3.70*E* + 02	1.31*E* + 02
F22	7.28*E* + 02	1.25*E* + 02	–	6.01*E* + 02	2.16*E* + 02
F23	5.50*E* + 02	3.37*E* + 01	=	5.60*E* + 02	6.57*E* – 01
+			1		
–			8		
=			12		

^*∗*^When six algorithms obtain the global optimum, the intermediate results are reported at *NFEs*=10000. “+,” “–,” and “=” denote that the performance of this algorithm is, respectively, better than, worse than, and similar to HDEMR at *α*=0.05.

**Table 4:** Results obtained by the multiple-problem Wilcoxon test for CEC2005 at *D*=10.

HDEMR vs.	*R* ^+^	*R* ^−^	*p* value	at *α*=0.05	at *α*=0.1
JADE	165.0	45.0	0.023907	+	+
CoDE	150.0	81.0	0.223788	=	=
jDE	190.0	41.0	0.009139	+	+
MPEDE	188.0	43.0	0.01117	+	+
SHADE	170.5	60.5	0.053725	=	+
LSHADE	139.5	70.5	0.191334	=	=
LSHADE-*ε*	164.5	66.5	0.085341	=	+

**Table 5:** Average rankings of contraction criterion combinations by the Friedman test.

*D*=10	
Parameters	Ranking
*E*_max_=1.0, *S*_max_=1.0	5.2857
*E*_max_=1.0, *S*_max_=2.0	4.6429
*E*_max_=1.0, *S*_max_=3.0	5.3095
*E*_max_=2.0, *S*_max_=1.0	4.6905
*E*_max_=2.0, *S*_max_=2.0	4.8333
*E*_max_=2.0, *S*_max_=3.0	5.1905
*E*_max_=3.0, *S*_max_=1.0	4.7143
*E*_max_=3.0, *S*_max_=2.0	4.4286
*E*_max_=3.0, *S*_max_=3.0	5.9048

**Table 6:** Average rankings of *C*_*max*_ by the Friedman test.

*D*=10	
Parameters	Ranking
*C*_max_=3	5.3333
*C*_max_=5	4.2619
*C*_max_=7	4.381
*C*_max_=9	3.3333
*C*_max_=11	3.6429
*C*_max_=13	3.5476
*C*_max_=15	3.5

**Table 7:** Comparison of mean error and standard deviation between HDEMR and other seven DE variants on CEC2014 at *D*=10.

Function	JADE			CoDE			jDE			MPEDE			SHADE			LSHADE			LSHADE-ε			HDEMR	
F01	0.00*E *+ 00	0.00*E* + 00	=	0.00*E* + 00	0.00*E* + 00	=	0.00*E* + 00	0.00*E* + 00	=	1.85*E* – 09	1.10*E* – 08	=	0.00*E* + 00	0.00*E* + 00	=	0.00*E* + 00	0.00*E* + 00	=	0.00*E* + 00	0.00*E* + 00	=	0.00*E* + 00	0.00*E* + 00
F02	2.09*E * – * *03	1.49*E* – 02	−	0.00*E* + 00	0.00*E* + 00	=	0.00*E* + 00	0.00*E* + 00	=	2.12*E* – 10	1.52*E* – 09	=	0.00*E* + 00	0.00*E* + 00	=	0.00*E* + 00	0.00*E* + 00	=	0.00*E* + 00	0.00*E* + 00	=	0.00*E* + 00	0.00*E* + 00
F03	8.74*E* – 03	2.79*E* – 02	−	0.00*E* + 00	0.00*E* + 00	=	0.00*E* + 00	0.00*E* + 00	=	0.00*E* + 00	0.00*E* + 00	=	0.00*E* + 00	0.00*E* + 00	=	0.00*E* + 00	0.00*E* + 00	=	0.00*E* + 00	0.00*E* + 00	=	0.00*E* + 00	0.00*E* + 00
F04	2.75*E* + 01	1.40*E* + 01	−	5.20*E* + 00	1.11*E* + 01	–	1.19*E* + 01	1.57*E* + 01	–	1.75*E* + 01	1.72*E* + 01	–	3.07*E* + 01	1.13*E* + 01	–	3.07*E* + 01	1.13*E* + 01	–	3.48*E* + 01	3.59*E* – 14	–	0.00*E* + 00	0.00*E* + 00
F05	1.75*E* + 01	4.87*E* + 00	+	1.96*E* + 01	2.80*E* + 00	–	1.89*E* + 01	3.20*E* + 00	+	1.67*E* + 01	4.58*E* + 00	=	1.62*E* + 01	6.97*E* + 00	+	1.55*E* + 01	8.00*E* + 00	+	1.17*E* + 01	9.29*E* + 00	=	1.96*E* + 01	2.81*E* + 00
F06	9.86*E* – 02	1.49*E* – 01	+	3.37*E* – 08	2.40*E* – 07	+	1.75*E* – 02	1.25*E* – 01	+	2.00*E* – 02	5.71*E* – 02	+	0.00*E* + 00	0.00*E* + 00	+	0.00*E* + 00	0.00*E* + 00	+	1.75*E* – 02	1.25*E* – 01	+	1.22*E* – 01	3.40*E* – 02
F07	1.29*E* – 02	8.51*E* – 03	−	3.91*E* – 02	2.35*E* – 02	–	1.82*E* – 02	1.34*E* – 02	–	1.81*E* – 02	1.52*E* – 02	–	4.51*E* – 03	6.52*E* – 03	+	2.62*E* – 03	5.90*E* – 03	+	5.85*E* – 04	2.39*E* – 03	+	9.22*E* – 03	7.62*E* – 03
F08	0.00*E* + 00	0.00*E* + 00	=	0.00*E* + 00	0.00*E* + 00	=	0.00*E* + 00	0.00*E* + 00	=	5.88*E* – 08	9.46*E* – 08	–	0.00*E* + 00	0.00*E* + 00	=	0.00*E* + 00	0.00*E* + 00	=	0.00*E* + 00	0.00*E* + 00	=	0.00*E* + 00	0.00*E* + 00
F09	3.41*E* + 00	8.69*E* – 01	−	4.02*E* + 00	2.18*E* + 00	–	5.66*E* + 00	1.35*E* + 00	–	6.52*E* + 00	1.95*E* + 00	–	3.07*E* + 00	8.13*E* – 01	–	2.37*E* + 00	8.48*E* – 01	=	1.86*E* + 00	6.89*E* – 01	=	2.97*E* + 00	1.69*E* + 00
F10	4.90*E* – 03	1.70*E* – 02	+	3.43*E* – 02	5.20*E* – 02	+	0.00*E* + 00	0.00*E* + 00	+	6.77*E* – 01	3.09*E* – 01	–	6.12*E* – 03	1.88*E* – 02	+	1.35*E* – 02	2.59*E* – 02	+	1.10*E* – 02	2.40*E* – 02	+	1.36*E* – 01	1.09*E* – 01
F11	9.15*E* + 01	5.94*E* + 01	=	8.29*E* + 01	1.00*E* + 02	=	2.69*E* + 02	1.02*E* + 02	–	2.83*E* + 02	1.14*E* + 02	–	7.79*E* + 01	6.22*E* + 01	=	2.24*E* + 01	2.30*E* + 01	+	1.71*E* + 01	1.59*E* + 01	+	1.08*E* + 02	9.63*E* + 01
F12	2.47*E* – 01	5.38*E* – 02	−	3.26*E* – 02	3.76*E* – 02	+	3.80*E* – 01	7.15*E* – 02	–	3.99*E* – 01	8.14*E* – 02	–	1.34*E* – 01	2.80*E* – 02	=	6.69*E* – 02	1.60*E* – 02	+	6.94*E* – 02	1.67*E* – 02	+	1.40*E* – 01	7.44*E* – 02
F13	8.61*E* – 02	1.68*E* – 02	−	8.66*E* – 02	3.52*E* – 02	–	1.42*E* – 01	2.78*E* – 02	–	1.27*E* – 01	2.37*E* – 02	–	7.29*E* – 02	1.43*E* – 02	–	4.74*E* – 02	1.29*E* – 02	–	4.41*E* – 02	1.64*E* – 02	–	1.81*E* – 02	8.76*E* – 03
F14	9.73*E* – 02	3.12*E* – 02	=	9.85*E* – 02	4.27*E* – 02	=	1.60*E* – 01	3.76*E* – 02	–	1.20*E* – 01	3.01*E* – 02	–	9.80*E* – 02	2.96*E* – 02	=	7.61*E* – 02	2.58*E* – 02	+	8.56*E* – 02	3.12*E* – 02	+	1.04*E* – 01	3.99*E* – 02
F15	5.56*E* – 01	9.46*E* – 02	−	6.03*E* – 01	1.78*E* – 01	–	1.02*E* + 00	1.57*E* – 01	–	9.40*E* – 01	1.67*E* – 01	–	4.84*E* – 01	7.81*E* – 02	–	3.89*E* – 01	7.33*E* – 02	+	3.73*E* – 01	7.10*E* – 02	+	4.40*E* – 01	1.18*E* – 01
F16	1.64*E* + 00	2.91*E* – 01	=	1.37*E* + 00	4.93*E* – 01	+	2.09*E* + 00	2.39*E* – 01	–	2.08*E* + 00	2.58*E* – 01	–	1.52*E* + 00	2.83*E* – 01	+	1.27*E* + 00	2.99*E* – 01	+	1.01*E* + 00	2.88*E* – 01	+	1.71*E* + 00	4.06*E* – 01
F17	5.18*E* + 01	3.30*E* + 02	−	2.46*E* + 00	4.78*E* + 00	+	9.89*E* + 00	1.08*E* + 01	=	2.92*E* + 01	1.02*E* + 01	–	4.62*E* + 00	1.42*E* + 01	+	8.95*E* – 01	8.11*E* – 01	+	3.12*E* + 01	4.91*E* + 01	–	6.80*E* + 00	5.51*E* + 00
F18	2.86*E* – 01	4.43*E* – 01	+	3.86*E* – 01	5.26*E* – 01	+	1.42*E* + 00	6.63*E* – 01	–	1.69*E* + 00	6.96*E* – 01	–	1.87*E* – 01	1.77*E* – 01	+	1.65*E* – 01	1.82*E* – 01	+	2.85*E* – 01	4.12*E* – 01	+	8.49*E* – 01	5.72*E* – 01
F19	2.65*E* – 01	7.72*E* – 02	−	7.49*E* – 02	5.15*E* – 02	+	3.56*E* – 01	1.25*E* – 01	–	4.50*E* – 01	1.55*E* – 01	–	2.64*E* – 01	2.63*E* – 01	–	8.76*E* – 02	8.34*E* – 02	+	2.85*E* – 01	4.06*E* – 01	–	1.33*E* – 01	1.44*E* – 01
F20	3.22*E* – 01	1.19*E* – 01	=	3.20*E* – 02	3.78*E* – 02	+	2.31*E* – 01	1.18*E* – 01	=	8.65*E* – 01	2.92*E* – 01	–	2.43*E* – 01	1.13*E* – 01	=	1.34*E* – 01	1.20*E* – 01	+	2.44*E* – 01	1.89*E* – 01	=	4.03*E* – 01	4.30*E* – 01
F21	1.60*E*+00	4.70*E*+00	−	1.74*E* – 01	1.95*E* – 01	+	3.77*E* – 01	2.65*E* – 01	–	3.15*E*+00	1.42*E*+00	–	3.62*E* – 01	2.60*E* – 01	=	3.87*E* – 01	2.49*E* – 01	–	2.64*E* + 00	8.84*E* + 00	–	2.63*E* – 01	2.23*E* – 01
F22	1.82*E* – 01	6.96*E* – 02	=	7.78*E* – 02	8.11*E* – 02	+	1.95*E* – 01	7.86*E* – 02	=	4.99*E* + 00	1.13*E* + 00	–	3.10*E* – 01	1.14*E* – 01	–	8.50*E* – 02	2.91*E* – 02	+	1.67*E* – 01	1.72*E* – 01	=	2.16*E* – 01	1.77*E* – 01
F23	3.29*E* + 02	2.87*E* – 13	−	3.29*E* + 02	2.87*E* – 13	–	3.29*E* + 02	2.87*E* – 13	–	3.29*E* + 02	2.87*E* – 13	–	3.29*E* + 02	2.87*E* – 13	–	3.29*E* + 02	2.87*E* – 13	–	2.00*E* + 02	0.00*E* + 00	=	2.00*E* + 02	0.00*E* + 00
F24	1.10*E* + 02	1.32*E* + 00	=	1.12*E* + 02	3.82*E* + 00	–	1.13*E* + 02	1.73*E* + 00	–	1.11*E* + 02	2.81*E* + 00	–	1.09*E* + 02	1.86*E* + 00	=	1.08*E* + 02	1.90*E* + 00	+	1.07*E* + 02	2.23*E* + 00	+	1.08*E* + 02	3.34*E* + 00
F25	1.30*E* + 02	2.99*E* + 01	=	1.35*E* + 02	3.50*E* + 01	=	1.36*E* + 02	3.50*E* + 01	=	1.24*E* + 02	1.05*E* + 01	=	1.38*E* + 02	4.14*E* + 01	–	1.41*E* + 02	4.51*E* + 01	–	1.38*E* + 02	3.78*E* + 01	=	1.25*E* + 02	1.64*E* + 01
F26	1.00*E* + 02	1.69*E* – 02	−	1.00*E* + 02	2.69*E* – 02	–	1.00*E* + 02	2.81*E* – 02	–	1.00*E* + 02	2.45*E* – 02	–	1.00*E* + 02	1.54*E* – 02	–	1.00*E* + 02	1.54*E* – 02	=	1.00*E* + 02	1.61*E* – 02	+	1.00*E* + 02	1.76*E* – 02
F27	1.09*E* + 02	1.60*E* + 02	=	6.25*E* + 01	1.42*E* + 02	–	7.43*E* + 01	1.41*E* + 02	–	2.28*E* + 00	4.56*E* – 01	+	1.50*E* + 02	1.68*E* + 02	=	5.41*E* + 01	1.25*E* + 02	–	6.31*E* + 01	9.51*E* + 01	–	2.10*E* + 01	7.13*E* + 01
F28	4.02*E* + 02	4.87*E* + 01	−	3.66*E* + 02	2.52*E* + 01	−	3.66*E* + 02	2.30*E* + 01	–	3.89*E* + 02	4.52*E* + 01	–	3.91*E* + 02	4.36*E* + 01	–	3.83*E* + 02	3.84*E* + 01	–	2.00*E* + 02	2.17*E* – 12	=	2.03*E* + 02	2.24*E* + 01
F29	2.47*E* + 02	4.14*E* + 01	−	2.20*E* + 02	1.26*E* + 01	−	2.21*E* + 02	6.46*E* + 00	–	2.22*E* + 02	7.75*E* – 02	–	2.22*E* + 02	6.35*E* – 01	–	2.22*E* + 02	5.30*E* – 01	–	2.00*E* + 02	0.00*E* + 00	=	1.99*E* + 02	1.86*E* + 01
F30	4.94*E* + 02	3.64*E* + 01	−	4.64*E* + 02	6.88*E* + 00	−	4.65*E* + 02	1.12*E* + 01	–	4.80*E* + 02	1.52*E* + 01	–	4.75*E* + 02	2.21*E* + 01	–	4.66*E* + 02	1.23*E* + 01	–	3.57*E* + 02	1.39*E* + 02	–	2.02*E* + 02	1.75*E* + 01
+			4			10			3			2			7			15			11		
–			16			13			19			23			12			9			7		
=			10			7			8			5			11			6			12		

“+,” “–,” and “=” denote that the performance of this algorithm is, respectively, better than, worse than, and similar to HDEMR according to the Wilcoxon rank-sum test at *α*=0.05.

**Table 8:** Results obtained by the multiple-problem Wilcoxon test for CEC2014 at *D*=10.

HDEMR vs.	*R* ^+^	*R* ^−^	*p* value	at *α*=0.05	at *α*=0.1
JADE	319.5	115.5	0.026666	+	+
CoDE	271.5	193.5	0.416534	=	=
jDE	375.0	60.0	0.000634	+	+
MP*E*DE	395.5	69.5	0.000771	+	+
SHADE	269.0	166.0	0.259551	=	=
LSHADE	219.5	245.5	1	=	=
LSHADE-*ε*	212.0	253.0	1	=	=

**Table 9:** GKLS test classes.

D	Hardness	*r*	*ρ*	Δ
2	Simple	0.9	0.2	10^−4^
2	Hard	0.9	0.1	10^−4^
3	Simple	0.66	0.2	10^−6^
3	Hard	0.9	0.2	10^−6^
4	Simple	0.66	0.2	10^−6^
4	Hard	0.9	0.2	10^−6^
5	Simple	0.66	0.3	10^−7^
5	Hard	0.66	0.2	10^−7^
10	Simple	0.66	0.3	10^−7^
10	Hard	0.66	0.2	10^−7^

For each test class, the dimension of test class (D), the radius of the convergence region *ρ*, distance betwen the paraboloid vertex and the global minimizer (*r*), and the tolerance Δ are given.

**Table 10:** Results of the experiments.

D	Class	HDEMR (10000 runs for the algorithm and class)	DIRECT (100 runs for the algorithm and class)
2	Simple	>1028.3(6)	197.26(0)
2	Hard	>17989(168)	1054.58(0)
3	Simple	>7569.9(67)	960.78(0)
3	Hard	>28182(268)	3651.54(0)
4	Simple	>80799(797)	>27608.34(2)
4	Hard	>351150(3505)	>112790.53(6)
5	Simple	>81913(811)	>15858.45(1)
5	Hard	>444170(4434)	>226665.45(17)
10	Simple	>71482(712)	>22067.58(2)
10	Hard	>60191(599)	>21774.72(2)

For each test class the average number of trails (or function evaluations) required to solve all 100 problems is presented for DIRECT algorithm. For HDEMR, the average number of trails (or function evaluations) required to solve each problem on 100 runs has been calculated, and the average of these 100 values is presented. The record “>*m*(*i*)” means that the algorithm does not solve a global optimization problem *i* times in 100 runs × 100 problems (i.e., in 10,000 runs for HDEMR and in 100 runs for DIRECT). In this case, the maximal number of trails (or function evaluations) set to 10^6^ is used to calculate the average number of trails (or function evaluations) *m*.

**Table 11:** The weight results of information literacy assessment index at CUG by HDEMR.

First-level index	Mean Value	Standard Deviation	Second-level index	Mean Value	Standard Deviation
L1	0.1958	0.0029	L11	0.4031	0.0008
			L12	0.2988	0.0006
			L13	0.2984	0.0006

L2	0.2085	0.0031	L21	0.2032	0.0006
			L22	0.3981	0.0011
			L23	0.4000	0.0012

L3	0.3115	0.0046	L31	0.0989	0.0014
			L32	0.3042	0.0043
			L33	0.3081	0.0043
			L34	0.3001	0.0042

L4	0.2958	0.0044	L41	0.4993	0.0018
			L42	0.2983	0.0011
			L43	0.2045	0.0007

## Data Availability

The data used to support the findings of this study are available from the corresponding author upon request.
